# Binding Costs in Processing Efficiency as Determinants of Cognitive Ability

**DOI:** 10.3390/jintelligence9020018

**Published:** 2021-04-01

**Authors:** Benjamin Goecke, Florian Schmitz, Oliver Wilhelm

**Affiliations:** Institute for Psychology and Pedagogy, Ulm University, Albert-Einstein-Allee 47, 89081 Ulm, Germany; florian.schmitz@uni-due.de (F.S.); oliver.wilhelm@uni-ulm.de (O.W.)

**Keywords:** mental speed, binding, working memory capacity

## Abstract

Performance in elementary cognitive tasks is moderately correlated with fluid intelligence and working memory capacity. These correlations are higher for more complex tasks, presumably due to increased demands on working memory capacity. In accordance with the binding hypothesis, which states that working memory capacity reflects the limit of a person’s ability to establish and maintain temporary bindings (e.g., relations between items or relations between items and their context), we manipulated binding requirements (i.e., 2, 4, and 6 relations) in three choice reaction time paradigms (i.e., two comparison tasks, two change detection tasks, and two substitution tasks) measuring mental speed. Response time distributions of 115 participants were analyzed with the diffusion model. Higher binding requirements resulted in generally reduced efficiency of information processing, as indicated by lower drift rates. Additionally, we fitted bi-factor confirmatory factor analysis to the elementary cognitive tasks to separate basal speed and binding requirements of the employed tasks to quantify their specific contributions to working memory capacity, as measured by Recall−1-Back tasks. A latent factor capturing individual differences in binding was incrementally predictive of working memory capacity, over and above a general factor capturing speed. These results indicate that the relation between reaction time tasks and working memory capacity hinges on the complexity of the reaction time tasks. We conclude that binding requirements and, therefore, demands on working memory capacity offer a satisfactory account of task complexity that accounts for a large portion of individual differences in ability.

## 1. Introduction

Some people process information fast, others do it more slowly. Although it is yesterday’s news that the processing efficiency of information is subject to individual differences (e.g., Carroll 1993; Roberts and Stankov 1999), there is still debate as to how individual differences in processing efficiency correspond to differential levels in human cognitive ability. On the one hand, empirically, it is a well-established finding that measures of mental speed correlate moderately with measures of cognitive ability (Sheppard and Vernon 2008). Further, this correlation becomes stronger as the complexity of the mental speed tasks increases. On the other hand, however, this well-replicated moderation by task complexity is not well understood (Deary et al. 2001; Sheppard and Vernon 2008). In fact, there is not even a satisfactory and theory-driven account of “task complexity”. Presumably, the most prominent proposition of “task complexity” is in terms of requirements on working memory capacity (WMC; e.g., Larson et al. 1988).

In the current study, we addressed this proposition by disentangling the underlying cognitive requirements of several mental speed tasks into basal requirements of mental speed and an incremental requirement on WMC in line with the binding hypothesis of WMC (Oberauer 2005a). This study aimed to contribute to a better understanding of task complexity in mental speed tasks. To this end, we combined theory-driven experimental manipulations with cognitive modeling and the analysis of individual differences. In the next sections, we embed mental speed into the framework of cognitive abilities and describe its typical mode of measurement. In addition, we explore how the complexity of elementary cognitive tasks can be understood in terms of WMC, before presenting the research aims of the current study.

### 1.1. Mental Speed as a Cognitive Ability and Its Measurement

The study of mental speed has a long tradition in psychology, dating back to the very beginnings of research on individual differences in cognitive ability (Galton 1883). The theoretical and empirical association between mental speed and cognitive ability is nowadays still evident in all broad models of intelligence that comprise broad mental speed factors, starting with the Gf–Gc theory (Cattell 1963) and continuing in the Three-Stratum theory (Carroll 1993) and Cattell–Horn–Carroll (CHC) theory (McGrew 2005, 2009). Generally, mental speed is understood as the ability to solve simple tasks quickly, i.e., to give as many correct responses as possible in a predefined time, or put differently, to carry out supposedly simple mental processes efficiently in a given time (Carroll 1993; Danthiir et al. 2005a). In this paper, we refer to mental speed as “the ability to make elementary decisions and/or responses (simple reaction time) or one of several elementary decisions and/or responses (complex reaction time) at the onset of simple stimuli” (McGrew 2009, p. 6), focusing on the idea of mental speed being indicative of efficiently processing limited information.

There are numerous studies on mental speed, but its structure is still inconclusive (e.g., Danthiir et al. 2005a). In part, somewhat ambiguous interpretations and differences in the suggested taxonomic models may contribute to this situation (Carroll 1993; Danthiir et al. 2005a; McGrew 2009). Historically, two different approaches have been adopted in studying mental speed (Danthiir et al. 2005a; Roberts and Stankov 1999). One approach is the descriptive psychometric approach of mental speed that is primarily concerned with providing simple indicators for a group factor of intelligence. The other one, the explanatory approach, focuses on studying the relationship between diverse measures derived from so-called elementary tasks and psychometric indicators of intelligence (Danthiir et al. 2005a). Current taxonomies of human abilities (McGrew 2005, 2009) comprise two distinguishable speed factors, namely processing speed (Gs; also “clerical speed”) and reaction and decision speed (Gt; also “elementary cognitive speed”). While Gs refers to the ability to smoothly carry out overlearned cognitive tasks, elementary cognitive speed refers to the ability to classify presented stimuli by means of button presses in computerized paradigms. Both factors capture the communality in tasks originating from the psychometric and explanatory traditions, respectively. However, this factorial distinction seems, to a large extent, to reflect differences in the assessment methods (Schmitz and Wilhelm 2019).

Psychometric models of mental speed suggest a general factor of mental speed (e.g., Burns and Nettelbeck 2003; Hale and Jansen 1994; Neubauer et al. 2000; Neubauer and Bucik 1996). However, studies using a broader selection of mental speed tasks conceive mental speed as a multifaceted construct (O’Connor and Burns 2003; Roberts and Stankov 1999). From the latter perspective, mental speed is seen as a construct of specificity, depending on the class of tasks (e.g., modalities and types of tasks). Recent research synthesized both perspectives by providing evidence for a hierarchical model of mental speed, comprising a general factor and several task-specific factors (Danthiir et al. 2005b, 2012; Kranzler and Jensen 1991; Roberts and Stankov 1999; Schmitz and Wilhelm 2016). Consequently, a wide variety of tasks have been proposed to be suited for measuring mental speed, for both paper–pencil and computerized contexts (Danthiir et al. 2005b, 2012).

Mental speed is usually assessed by means of so-called elementary cognitive tasks (ECTs; Carroll 1993). These tasks are labeled “elementary” because they only require basal cognitive processes and no specific knowledge or previous experience. In fact, the assumed simplicity of these tasks is accentuated through the idea that every person should be able to solve the tasks correctly given enough time. The small number of mental processes that are to be carried out to arrive at the correct solution comes with the advantage that unwanted sources of individual differences are minimized, strategy use is prevented, and empirical control of task complexity is supposedly provided (Schubert et al. 2015). Although the cognitive demands of these tasks might be low, several cognitive processes are involved when completing ECTs: (sustained) attention; initial perception of stimuli; encoding, updating, and retrieval from working memory; response preparation; and execution of a motor response (Ackerman and Kyllonen 1991; Kyllonen and Christal 1990).

Due to the simplicity of ECTs, the error rates in these tasks are typically low and hypothesized to be randomly distributed across trials and individuals (Danthiir et al. 2005a). Apart from error rates, the most prominent variable of interest is response times, that is, the time it takes a person to give a (correct) response. Take, for example, a stimulus comparison task (Danthiir et al. 2012) with a binary decision format, where a person is simultaneously presented with two stimulus strings and is asked to determine whether these two strings are the same or different. This task is inherently simple, but persons will differ in their time to give the correct response, as some persons are able to process the stimuli faster than others are. The emerging response time distributions are suited for studying individual differences (Schmitz et al. 2018). Response time distributions of ECTs contain much information, which can be analyzed in detail. However, most studies used mean response times only as performance indicators (for an overview on scoring alternatives for mental speed tests, see Schmitz et al. 2018). In order to extract more information on response times on ECTs, reaction-time models like the diffusion model (Ratcliff 1978; Ratcliff and Rouder 1998) have become increasingly popular. The diffusion model’s basic idea is to decompose a binary decision process into well-defined parameters that may serve to indicate cognitive processes (Ratcliff 1978). For instance, the efficiency of information processing (i.e., drift rate) is separated from response caution. Additionally, non-decision time is quantified, which captures motor execution, among other parameters (for overviews, see Ratcliff and McKoon 2008; Voss et al. 2013; Wagenmakers 2009). Of these parameters, drift rates were shown to be the most relevant parameter capturing individual differences in task performance (e.g., Ratcliff et al. 2010, 2011; Schmiedek et al. 2007; Schmitz and Wilhelm 2016). One potential advantage of this performance modeling is that drift rates capture the information contained in the distributions of both correct and erroneous responses, controlling for individual differences in the speed–accuracy settings (Phillips and Rabbitt 1995).

### 1.2. Correlations of Performance on ECTs with Cognitive Abilities

Performance on ECTs has been studied in relation to individual differences in cognitive ability. In fact, reductionist theories of intelligence have postulated mental speed as fundamental for higher cognitive processes (Eysenck 1987; Jensen 1982, 2006; Nettelbeck 2011). This makes mental speed one of the explanatory candidate mechanisms for cognitive abilities, additionally to WMC (e.g., Chuderski et al. 2012; Kane et al. 2005; Kyllonen and Christal 1990; Oberauer et al. 2005). Further, it has even been claimed that mental speed also contributes to WMC, as the processing and rehearsal of information is dependent on a shared and time-dependent resource (i.e., time-based resource sharing model; Barrouillet et al. 2007, 2011).

From an empirical point of view, the notion that differential levels of performance in ECTs correspond to differential levels in cognitive abilities, such as reasoning and WMC, has been supported in research syntheses: two meta-analyses have shown that response times are consistently and moderately negatively correlated with cognitive abilities (Doebler and Scheffler 2016; Sheppard and Vernon 2008). Across 172 studies, Sheppard and Vernon (2008) found a mean correlation of *r* = −0.24, while Doebler and Scheffler (2016) found a range of correlations from *r* = −0.18—*r* = −0.28. Regarding the correspondence of mental speed with cognitive abilities, it is especially the proposed general factor of mental speed in hierarchical models, which shows the predictive validity of ability factors like Gf or WMC, whereas task-specific factors do not contribute to explaining variance in those constructs (Schmitz and Wilhelm 2016, 2019). In addition to correlations between response times as a performance index of ECTs and cognitive ability, several studies have reported associations for the drift rate of the diffusion model with measures of cognitive ability. For example, Schmiedek et al. (2007) used structural equation modeling to yield factors corresponding with the diffusion model’s parameters, hence the reliable shared variance in the parameter estimates. The drift rate factor was positively correlated with a broad working memory factor (*r* = 0.65). In line with this finding, several other studies found correlations between a drift rate factor and measures of cognitive ability, with mostly moderate correlations (Ratcliff et al. 2010, 2011; Schmitz and Wilhelm 2016), thus generally supporting that drift rate is suited to index individual differences in processing efficiency.

There is at least one moderating factor of this predictive validity: it is a well-replicated finding that ECTs correlate more strongly with cognitive ability as the complexity of the speed task increases (Sheppard and Vernon 2008). Although the increments of predictive validity towards cognitive ability might be small in magnitude (Deary 2003), this finding is known as the “complexity hypothesis” (Vernon and Weese 1993). These effects of task complexity (which might empirically be indicated by slower response times or lower accuracies) provoke questions: (1) what is “complexity” in tasks that should be elementary by definition? (2) How and by which means would complexity contribute to the increased correlations with cognitive ability? Typically, studies investigating the complexity hypothesis increased the task requirements of ECTs by increasing the bits of information to be processed (e.g., Roth 1964; Vernon and Weese 1993; Vernon and Jensen 1984; Marshalek et al. 1983). This requirement of ECTs to maintain several arbitrary bits of information in mind (e.g., stimulus–response (S–R) mapping rules) was hypothesized to put demands on WMC (Wilhelm and Oberauer 2006)—it has thus been ruled likely that WMC requirements in speed tasks are the driving factor for the increase in predictive validity for intelligence (Meiran and Shahar 2018). Hence, it can be hypothesized that individual differences in WMC contribute to the well-documented complexity moderation when predicting cognitive ability from mental speed tasks. Specifically, more complex ECTs should correlate more highly with cognitive abilities like reasoning or WMC than simple ECTs, as more complex ECTs put more demands on WMC, and are therefore more akin to tests of reasoning or WMC regarding their underlying cognitive demands (Kranzler and Jensen 1991; Marshalek et al. 1983).

### 1.3. Working Memory Capacity and Complexity in ECTs

Working memory can be defined as a cognitive construct, which is used to “mentally maintain information in an active and readily-accessible state, while concurrently and selectively processing new information” (Conway et al. 2008, p. 3). It can be understood as a system that enables more complex cognitive processes, like reasoning, problem-solving, and decision making (Wilhelm et al. 2013), but also language comprehension and planning (Cowan 2005). The system simultaneously maintains relevant information and grants access to prior acquired information, both of which are required for these cognitive processes. In addition, it is assumed to store information and control cognitive processes (Miyake and Shah 1999).

Theories of working memory share one vital notion, namely, that it has limited capacity. That is, the possible amount of information that can be stored and processed in working memory is limited (Baddeley 2012; Conway et al. 2008; Cowan 2005). This limitation is reflected in the term “working memory capacity” (WMC), which has also been used to describe individual differences (Cowan 2010; Wilhelm et al. 2013). Accordingly, capacity limitations in working memory have been theorized to account for performance restriction in cognitive tasks like reasoning and decision making. Results show that persons with a lower capacity are outperformed by individuals with higher capacity in these tasks (Wilhelm et al. 2013). Accordingly, WMC has been found to be strongly related with reasoning ability (Kane et al. 2005; Kyllonen and Christal 1990; Oberauer et al. 2005) and discussed as constituting the very core of reasoning ability (e.g., Kyllonen and Christal 1990). Hence, it can be theorized that WMC plays a central role for the observed associations between speed and cognitive ability.

With respect to the possible role of WMC in completing ECTs, we would like to point out two requirements of ECTs. First, all ECTs require fast responses, and second, most ECTs require maintaining a number of task-relevant bits of information. While the former requirements translate into a basal speed requirement, the latter might, for instance, comprise stimulus–response mapping rules (for example, in a substitution task), which need to be maintained in working memory. Both the basal speed affordance and the incremental WMC requirement might contribute to explaining variance in human cognitive abilities.

Referring to the first and more basal requirement of ECTs, mental speed is assumed to play a causal role for differential levels of all cognitive functions and be causal for correlations between WMC and intelligence by some researchers (Kail and Salthouse 1994). Basically, mental speed should determine the efficiency of processing elementary cognitive operations and information. Thus, the faster and more efficient a person is, the better their cognitive performance. The time-based resource-sharing model (Barrouillet et al. 2007, 2011) is one such theory. According to this account, individuals who are able to process information faster (i.e., more efficiently) have more time for rehearsing the information and therefore show higher levels of WMC. The time-based resource-sharing model predicts that memory sources decay as a function of time. Assuming that an executive resource is used for both processing and rehearsal, it can be predicted that faster processing allows for more time for rehearsing information, thereby counteracting decay and increasing WMC (but see Lewandowsky et al. 2009 for a critical discussion).

Regarding the second requirement—and therefore referring to the complexity of ECTs—working memory can be conceptualized as a system of relations, as proposed by the binding hypothesis of WMC (Oberauer 2005a, 2019; Wilhelm et al. 2013). According to the binding hypothesis, WMC reflects the limit of a person’s ability to establish, maintain, and update temporary and arbitrary bindings (e.g., relations between items or relations between items and their context, new order of words, new spatial arrangements of known objects, etc.). Therefore, it was hypothesized that the limited capacity of working memory is the result of interference between diverse bindings (Oberauer et al. 2008; Wilhelm et al. 2013). Given that the capacity of bindings is limited, people can only relate a limited number of propositions, thereby also limiting reasoning ability (Oberauer et al. 2008). Two lines of research have supported this particular account of WMC. First, tasks assessing the ability to build and maintain structural relations have been shown to be correlated with classical WMC measures and tests of reasoning ability (Oberauer et al. 2008; Wilhelm and Oberauer 2006), although there are studies that provide less evidence in this regard (Hülür et al. 2019). Further, it has been shown that WMC limits memory for bindings, rather than for single items (Oberauer 2019). Second, it has been demonstrated that only recollection is correlated with WMC (Oberauer 2005b), suggesting a particular role for relational retrieval.

Both accounts of WMC, mental speed, and binding capacity propose an account of individual differences in cognitive abilities. For the time-based resource-sharing model, it is the mere time that elapses between encoding and retrieving information, while for the binding hypothesis, it might be both the interference between but also the mere maintenance of several diverse bindings that are crucial for the limitation of WMC. Importantly, we predict that both aspects of working memory contribute to performance in ECTs and their relation with cognitive ability. That said, basal speed may be the crucial factor in simple ECTs. However, as task complexity is increased in ECTs (e.g., more S–R mapping rules), so is the demand on working memory (e.g., binding capacity). Consequently, WMC requirements in complex ECTs can be assumed to be a causal factor incrementally contributing to the relation with cognitive ability.

### 1.4. The Present Study

The aim of this study was to replicate and extend previous research addressing the task complexity moderation of the relationship of mental speed with cognitive ability. Using a well-defined theoretical binding framework, we intended to offer a more meaningful account of “task complexity”. To this end, we explicitly manipulated task complexity in three mental speed tasks. In their basic form, all tasks fulfilled the requirements of ECTs in terms of task simplicity. We then systematically manipulated WMC requirements in terms of bindings in two conditions. To do so, we increased the number of task-relevant stimulus–stimulus (S–S) or stimulus–response (S–R) bindings that participants needed to maintain in an active state while performing the tasks. The manipulation allowed for a targeted isolation of task complexity. To this end, we aimed at identifying the WMC requirement condition, which shows the most substantial effects of the experimental manipulations in terms of mean effects in response times and errors.

We intended to show that increasing WMC requirements by virtue of manipulating the number of bindings strengthens relations with WMC over and above mental speed. Specifically, we hypothesized that:

(1) increasing binding requirements results in higher task complexity which would be reflected by more effortful information processing in the difficult conditions of each ECT as contrasted with the easier conditions (slower response times, lower accuracies, and lower diffusion model drift rates);
(2) as binding requirements were manipulated in each ECT, this constitutes an analogous increase in WM requirements. In turn, this constitutes a WMC-related communality which can be modeled as a specific factor across tasks;
(3) the WMC-related specific factor is incrementally predictive of cognitive ability over and above basal speed. This implies that the predictiveness of complex ECTs is partly driven by WMC contributions to performance.

## 2. Methods and Materials

### 2.1. Participants and Procedure

The present study was advertised both with flyers and on social media, and data were collected in four German cities. Participants had to be between 18 and 35 years and fluent in German. Data were collected in groups with up to eight persons at a time. Tasks and instructions were administered by computer. All tasks were presented on identical 14″ notebooks and controlled by compiled C++ programs using SDL libraries for stimulus timing and response collection. Participants completed tasks in the same order, and trial lists were presented in a pseudo-random way, meaning that the trialists were randomized before presentation. Responses were given by pressing the left or right Ctrl key, respectively, if not specified differently, and participants were instructed to keep their index fingers on the two response keys. Standardized instructions were provided on screen. A trained proctor supervised the session and provided instructions if requested. In between the tasks, participants had two 5 min breaks during which we provided snacks and something to drink. In total, *n* = 127 participants were recruited. We did not determine the sample size in advance, but rather collected data until a meaningful sample size for modeling purposes was reached (e.g., similar in size as in Wilhelm and Oberauer 2006 or Hülür et al. 2019). A local ethics committee approved the data collection, and all participants provided informed consent prior to participation. After completing the 2.5 h computerized test battery, they were compensated with EUR 20 or partial course credit. The mean age of the sample was *M* = 22.1 years (*SD* = 4), and 72.4% were female. The majority of participants (*n =* 120) were highly educated, holding at least a high school degree. Almost all (*n =* 124) indicated they were native German speakers.

### 2.2. Measures

#### 2.2.1. Speed Tasks

**Figure 1:**
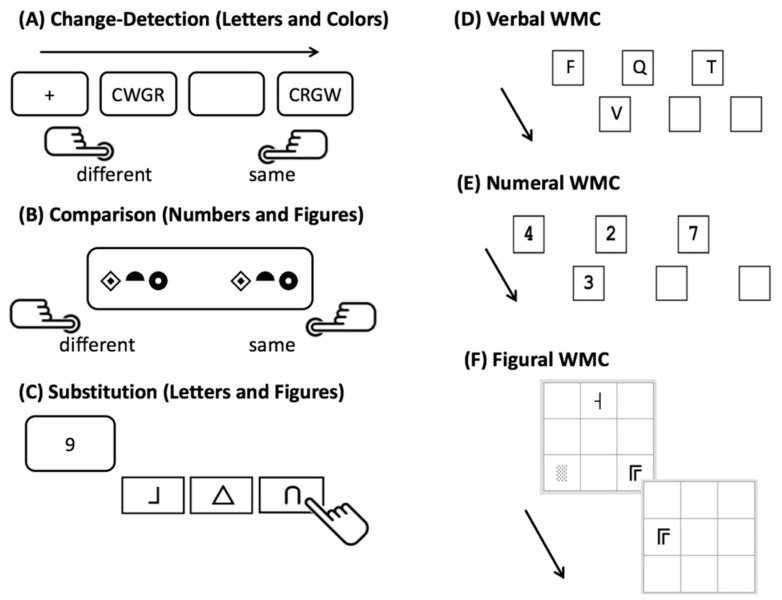
Overview of mental speed tasks (**A**–**C**), and of working memory capacity (WMC) tasks (**D**–**F**). Each mental speed task was presented with the set sizes of 2, 4, and 6 stimuli per trial, respectively.

For the test sessions, we used three computerized speed tasks: a change-detection task (Luck and Vogel 1997; Rouder et al. 2011), a stimulus comparison task (Danthiir et al. 2012), and a substitution task (Danthiir et al. 2012) (see Figure 1 for a schematic overview). Each task was administered with two different sets of stimulus materials in three different set sizes (i.e., the presented numbers of stimuli, namely 2, 4, and 6). The set sizes (requiring stimulus–stimulus or stimulus–response bindings) were manipulated in order to increase the WMC load of the tasks in line with the binding hypothesis.

The change-detection task was used with either color (30 equidistant color tones on a color circle, with comparable luminescence) or letters (20 capitalized consonants, excluding “Y”) as stimuli. Each trial started with the presentation of a fixation cross for 400 ms. Next, the stimuli were shown in the form of a horizontal string on the screen. The presentation time was 125 ms per stimulus. After the presentation of the stimulus string, participants were presented with a blank screen for 1000 ms and subsequently with a new stimulus string of the same length and the same stimulus modality as the first string. Participants had to indicate whether all elements of the first and second string were identical or whether some elements swapped their positions. The ratio of change vs. non-change trials was 50:50. Response time was not limited. The change-detection task consisted of 2 parts with 3 blocks each. Blocks contained 44 trials each (2 warm-up trials and 42 trials used for the analyses). Overall, there were 84 trials per set-size condition. Prior to the testing phase, participants had to complete 18 practice trials. Inter-trial intervals (blank screens) were displayed for 100 ms.

The comparison task was administered with 18 different abstract figures or digits (1–9) as stimuli. As in the change-detection task, 2, 4, or 6 stimuli could be used in each task variant. Generally, the administration of the comparison task resembled the administration of the change-detection task, with the difference that the two strings of stimuli were shown simultaneously on the screen, aligned horizontally. Prior to the presentation of the two stimulus strings, two fixation crosses were displayed for 400 ms at the respective positions where the stimuli would appear. Participants had to indicate whether the strings of stimuli were exactly identical or different by pressing the corresponding buttons on the keyboard. In the case of a difference, two elements swapped position. Stimuli remained on screen until participants responded. The numbers of blocks and trials, including the number of practice trials, for the comparison task were the same as those in the change-detection task. For the change-detection and the comparison task, the set sizes (2 stimuli, 4 stimuli, and 6 stimuli per string), the proportion of same/different stimulus pairs, and the position swaps were balanced across all trials.

For the substitution task, different abstract figures and letters were used as stimuli. In this task, stimuli were arbitrarily mapped from one stimulus domain to another. For instance, figures were mapped with colors, and the letters were mapped with numbers. Depending on the condition, mappings comprised two, four, or six stimulus-response relations, respectively. Prior to the task, participants had to learn the instructed stimulus-response mappings and practice them in a practice phase prior to the testing phase. The task consisted of 6 parts (3 parts per modality per condition two, four, or six), each of which consisted of 5 blocks with 26 trials (2 warm-up trials and 24 trials used for analyses). Overall, there were 96 test trials for each complexity condition for both modalities. In each trial of the substitution task, one stimulus was presented on screen, and the participant had to respond to that stimulus by pressing a key indicating the respective response stimulus. The number keys in the top row of the keyboard were used as response keys and special templates were fixed to them, displaying the response domain’s respective stimuli. After eliciting a response, participants were asked to press the spacebar with the index finger of their dominant hand in order to initiate the subsequent trial. Once the next stimulus appeared, they had to press the corresponding button in the upper row on the keyboard with their index finger. Again, trial requirements, stimuli, and trial-type order were balanced in this task, and participants were presented with 18 practice trials.

#### 2.2.2. Working Memory Capacity

We used three Recall−1-Back (R1B) tasks, which have been shown to be psychometrically satisfactory indicators of WMC (Wilhelm et al. 2013). These tasks were designed to measure the recall of continuously updated stimuli (see Figure 1). They were constructed following a matrix design, comprising different combinations of memory load and required updates. Task requirements varied from block to block in terms of working memory load (1, 2, 3, or 4 stimuli to remember) and updating requirements (6, 9, or 12 updates). First, participants completed 27 practice trials. Participants then completed 12 blocks (with a total of 108 responses) in the test phase. For the scoring of the R1B tasks, we applied partial credit scoring, i.e., the proportion of correct responses in all trials (Conway et al. 2005), which has been shown to yield reliable scores of task performance.

In the verbal task version, two, three, or four letters (corresponding to three possible load conditions) were initially presented in two, three, or four separate boxes on the screen, depending on the load level of the corresponding trial. Stimuli were presented in boxes horizontally aligned in the center of the monitor and were displayed throughout the entire run, including the 500 ms inter-trial interval. With every trial, a new letter appeared in one of the boxes and the participants were asked to type in on the keyboard the letter previously presented in this particular box. Participants were instructed to type in the previously shown letter as long as the new letter was shown (3000 ms presentation time). Responses entered after this period were treated as errors. Prior to the test trials, participants completed 27 practice trials with one, two, or three boxes to familiarize themselves with the procedure. In these practice trials, participants were given trial-wise feedback about the accuracy of their responses. However, feedback was not provided during the testing phase. All trials were balanced regarding positions and repetitions of stimuli. The same procedure and design were used in the numerical R1B task, with the only difference being that numbers from 1–9 were used instead of letters. In contrast to the previous tasks, a 3 × 3 grid was shown in the figural variant of the R1B task. Simple symbols (size: 79 pt × 79 pt) were presented in randomly selected cells (size: 150 pt × 150 pt) of the grid. Participants indicated by mouse click the position in the grid in which the currently displayed stimulus had been shown when it was shown the previous time. As in the verbal and numeral variant, participants had to respond within a window of 3 s during which the current stimulus was visible.

The final task order was: SUB.fig—part 1, CMP.num—part 1, SUB.fig—part 2, CDT.col—part 1, SUB.fig—part 3, CMP.num—part 2, CDT.col—part 2, R1B.num, break (5 min), SUB.let—part 1, CMP.fig—part 1, SUB.let—part 2, CDT.let—part 1, SUB.let—part 3, R1B.fig, break (5 min), CDT.let—part 2, CMP.fig—part 2, R1B.let, demographical questionnaire.

### 2.3. Statistical Analysis

#### 2.3.1. Data Treatment

Prior to statistical analysis, the data were carefully checked for outliers and implausible values. To this end, the raw data of *n* =127 participants were treated in four steps. First, we removed all warm-up trials from the data and then applied the liberal Tukey criterion (Tukey 1977), i.e., responses were excluded when they were more extreme than 3 interquartile ranges either above the 75th percentile of the RT distribution or below the 25th percentile, or below 200 ms. Second, we excluded participants with frequent missing values (>30) in more than one task (*n* = 12), leaving *n* = 115 for the analyses. Next, we computed task scores. Finally, missing values on the task score level were replaced using multiple imputations via the *R*-package *Amelia* (Honaker et al. 2011), which uses a maximum likelihood estimator (expectation maximization) to impute the missing data points. For the comparison and change-detection tasks, no missing data points had to be imputed, while for the substitution and the Recall−1-Back tasks, less than 0.05% of cells were imputed.

#### 2.3.2. Scoring of ECT Performance

Several scores were computed for the data, as frequently done when analyzing RT data obtained in elementary cognitive tasks (e.g., Heathcote et al. 1991; Hohle 1965), including response times and the error rates (i.e., accuracies). The mean latency of correct responses is frequently used in laboratory research, as it depicts a parsimonious indicator of the performance on ECTs, with faster response times indicating better processing efficiency (e.g., Danthiir et al. 2005a). In turn, error rates might reflect cognitive slips, lapses, or mistakes, although errors are rare events in ECTs, supposedly due to their simplicity, and thus error rates exhibit low variability across persons. Both scores might be biased. For example, the error rates could suffer from individual differences regarding speed–accuracy trade-offs, where participants either sacrifice speed for accuracy or accuracy for speed in responses to gain momentum. Although task performance in ECTs is usually reported in terms of response times, we expected that the manipulation of task complexity also affected error rates, as usually observed when administering conventional measures of WMC (e.g., Conway et al. 2005).

Generally, we expected to find effects of task complexity in both the mean response times and the mean error rates across the complexity conditions within the task classes (Lohman 1989). However, as both indicators are prone to individual speed–accuracy trade-offs, it remains unclear whether these indicators are suitable to depict the effects of task complexity across persons in a comparable fashion. Usually, faster response times are associated with higher levels of cognitive abilities (Doebler and Scheffler 2016; Sheppard and Vernon 2008). According to the complexity hypothesis, this correlation is increased with elevated task complexity (Sheppard and Vernon 2008; Vernon and Jensen 1984). At the same time, however, increasing task complexity is paralleled with higher error rates, which in turn is then also significantly correlated with intelligence (Vigneau et al. 2002). The desired information of cognitive processing is intrinsically part of both indicators, and thus it is necessary to find a joint parameter bringing both types of information together.

In order to capture performance information in both the response times and the error rates, and additionally to control for individual differences in the speed–accuracy trade-off, we analyzed the data with a simplified diffusion model (e.g., Ratcliff 1978). Specifically, we employed the R package EZ (Wagenmakers et al. 2007), which uses a closed-form expression yielding scores corresponding with the 3 most relevant parameters of the diffusion model: the drift rate (*v*), the response criterion (*a*), and the non-decision time (Ter). The EZ algorithms have been shown to be robust for the modeling of individual differences even when only limited trial numbers are available (Ratcliff and Childers 2015; van Ravenzwaaij and Oberauer 2009), like in the present study.

The diffusion model offers an account for modeling cognitive processes in binary decision tasks. The drift rate corresponds with the mean rate at which an information accumulation process reaches the correct response boundary. This parameter reflects person ability (e.g., Schmiedek et al. 2007). In turn, the response criterion denotes the separation of the response boundaries (thresholds) and corresponds with the speed–accuracy trade-off. Depending on how cautious a person is, performance on ECTs could be reflected in either response times or error rates, or both. The diffusion model accounts and controls for such differences in response caution and yields the drift rate parameter, which is hoped to be a less biased measure of task performance and has been shown to be a valid predictor of WMC and intelligence (Schmiedek et al. 2007; Schmitz and Wilhelm 2016). Finally, the third parameter, non-decision time, is thought to depict the time beyond the actual decision and is conventionally interpreted as the time required for motor execution and stimulus encoding.

Parameters of the EZ diffusion model could be computed for all tasks with a binary response format. As the substitution tasks required pressing up to 6 response keys, we calculated an alternative performance score capturing the information in speed and accuracy. Specifically, we computed a composite score reflecting the mean standardized reciprocal response times (1/RT) and the mean standardized relative accuracy (1-error rate). Such a composite score has been successfully used in other studies comprising speeded response time tasks (e.g., Stahl et al. 2014).

#### 2.3.3. Structural Equation Modeling

In order to disentangle speed and binding requirements of the ECTs, we fit bi-factor models to the data (e.g., Eid et al. 2008), specifying nested factors capturing the effects of the binding manipulation. For the comparison and the change-detection task, we used the respective drift rates as indicators, while for the substitution task, we used the above-described compound scores as indicators. The factors were identified using effects coding (Little et al. 2006). The following fit indices were considered as an indication for good model fit: comparative fit index (CFI) ≥ 0.95, root mean square error of approximation (RMSEA) ≤ 0.06, and standardized root mean square residual (SRMR) ≤ 0.08 (Hu and Bentler 1999). In addition to that, the following indices were considered for acceptable model fit: CFI ≥ 0.90; RMSEA < 0.08, and SRMR ≤ 0.10. Prior to modeling, indicator variables were standardized.

For all statistical analyses, we used R (R Core Team 2020). We used the psych package (Revelle 2020) for standard psychometric analyses, the effsize package (Torchiano 2020) for effect sizes and the lavaan (Rosseel 2012) and the semTools packages (Jorgensen et al. 2020) for the confirmatory factor analysis and structural equation modeling. Scripts and data are provided in an online repository: https://osf.io/em3sg/ (accessed on 23 March 2021).

## 3. Results

In Table 1, we report descriptive statistics—including mean response times, mean error rates, mean compound scores (for substitution tasks), and diffusion model parameters. Further, effect sizes (Cohen’s *d*; Cohen 1969) are given for differences in response times and error rates between experimental conditions (set sizes 4 and 6, respectively, vs. set size 2) for all tasks. Compliant with our expectations, we observed strong effects for the complexity manipulations on the mean response times of all task classes. Across all task classes, participants tended to respond slower as the complexity of the tasks increased. All standardized mean differences exceeded a value of *d* ≥ 1, indicating that participants responded more than one standard deviation slower in task conditions with increased binding requirements. As expected, the effects for the complexity manipulations were most vital for the tasks administering set size 6.

In addition to the slowing in response times, the complexity manipulations generally resulted in increased error rates. However, results were less consistent here. For both change-detection tasks (i.e., color and letter), the error rates consistently increased with increasing task complexity, in particular in set size 6 condition. Error rates were also increased in the comparison tasks (i.e., figure and number), but only the figural variant had a substantial increase in error rate at set size 6. For the numerical task variant, the effect of the complexity manipulation for set size 4 vs. set size 6 was comparable. Lastly, descriptive results for the substitution tasks looked similar. Although the standardized mean differences between the error rates were increased to a lesser extent compared to the other two task classes, the effects were still noticeable. For the substitution task with figural stimuli, the mean error rates were more strongly increased in the more complex condition (set size 6), while for the substitution tasks with letters as stimuli, the effects of set size 4 and set size 6 were again comparable. Overall, the effects in the error rates were not as strong as in response times, but still substantial, especially for the more complex (i.e., set size 6) condition.

We also expected the parameters of the diffusion model to be affected by the experimental manipulations. As can be seen in Table 1, with increasing complexity of the tasks, drift rate (v) decreased for all tasks, suggesting decreasing processing efficiencies due to elevated task affordances (e.g., encoding more stimuli into working memory). In addition, the non-decision time (Ter) increased across the two binary response format task classes (i.e., comparison and change-detection). Also, the response caution (a) increased with elevated complexity affordances for the comparison but only to a lower degree for the change detection tasks.

Given that the most substantial effects of the experimental manipulations were observed in the set size 6 condition, we chose this condition as the high-WMC requirement condition in addition to the basal set size 2 condition for the latent modeling approach. The difference in processing efficacy between both set sizes (i.e., 2 vs. 6) was strong enough to assume that the complexity manipulation was successful and yielded sufficient power for the intended analyses.

**Figure 2:**
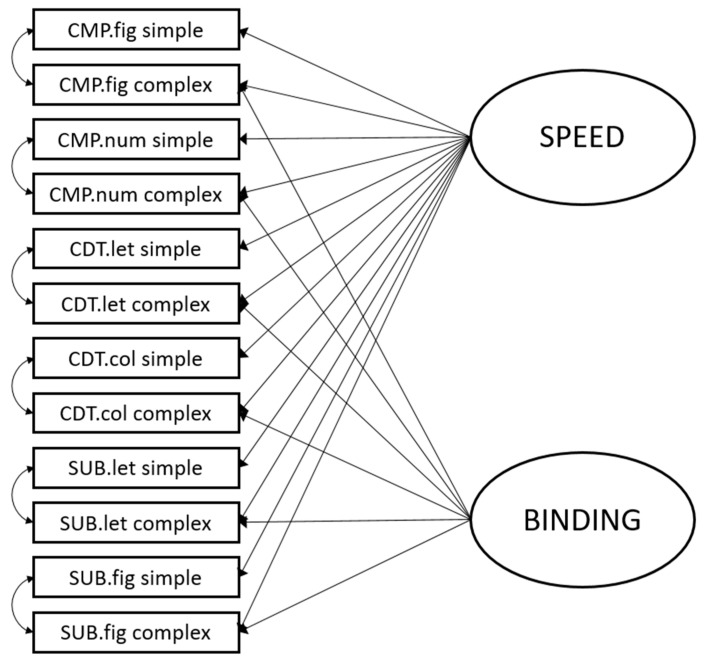
Schematic measurement model of speed and binding. CMP.fig = comparison figural, CMP.num = comparison numerical numerical, CDT.let = change detection letter, CDT.col = change detection color, SUB.let = substitution letter, SUB.fig = substitution figure.

To test whether it was possible to disentangle basal speed and increased complexity requirements (i.e., binding) in the administered ECTs, we first report the fit indices of a bi-factor measurement model. This model followed one specific construction rationale: capturing the communality of the speed requirements in the administered tasks by means of a general factor and capturing the remaining variance in a nested factor accounting for the elevated binding requirements of the tasks. The measurement model was specified using the respective indicators of set sizes 2 and 6—put differently, the simple and the complex indicators. Therefore, the measurement model included 12 indicators (3 tasks × 2 modalities × 2 complexities). All indicators were loaded on a general factor, capturing the basal speed requirement of the administered tasks. For this factor, all loadings were estimated freely. In addition to that, the more complex tasks were also loaded on a nested factor, capturing the remaining variance for the increased binding requirements of the tasks. In order to account for task specificities, correlated residuals were allowed between the simple indicators of a task variant and the more complex indicators of a particular task. The model is depicted in Figure 2. It yielded an acceptable fit for the data: *n* = 115, *χ*^2^(41) = 74.33, CFI = 0.923, RMSEA = 0.084, SRMR = 0.058. This model fit the data significantly better than a single-factor model, not accounting for binding requirements (∆*χ*^2^(7, *n* = 115) = 23.09, *p* < 0.05). The loadings are depicted in Table 2; they were all significantly larger than zero on both factors. The general speed factor accounted for a substantial proportion of variance in the indicators (*ω* = 0.83; McDonald 1999), while the nested binding factor accounted for a smaller yet substantial proportion of variance in its indicators (*ω* = 0.40). As both factors had significant variance (*φ*_Speed_ = 0.24, *p* < 0.001; *φ*_Binding_ = 0.06, *p* < 0.01), both a general factor reflecting individual differences in speed and a specific factor capturing individual differences in binding ability were confirmed. Therefore, binding requirements can be dissociated from basal speed requirements.

**Figure 3:**
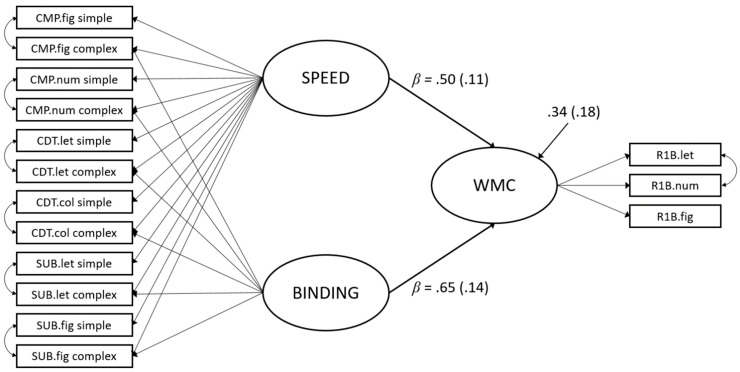
Latent regression model with speed and binding predicting working memory capacity (WMC). Parameter estimates are fully standardized and standard errors are given in parentheses.

Finally, we tested the predictive validities of the identified factors for the measurement models. In order to do so, a latent factor for WMC was added to the model and regressed on the general speed and the nested binding factor. Goodness of fit statistics for the measurement model of WMC are not provided, as this model was only comprised of three indicators and therefore just identified. The specification of the measurement model was not altered. For the measurement model of WMC, the residuals of the two indicators using stimuli that can be represented phonologically (i.e., letters and numbers) were allowed to correlate. This model, including the standardized regression weights (betas), is depicted in Figure 3. Details of the measurement models (i.e., loadings) are given in Table 2.

The structural model depicting the complexity manipulation of 2 vs. 6 stimuli per task exhibited good overall model fit: *n* = 115, χ^2^(74) = 100.81, CFI = 0.949, RMSEA = 0.056, SRMR = 0.060. The predictors explained 66.5% of the variance in WMC. All loadings were significantly larger than zero (see Table 2). The factor saturations associated with the speed factor, the binding factor, and the WMC factor were ω = 0.83, ω = 0.46, and ω = 0.57, respectively. Both exogenous factors had significant variance (*φ_Speed_* = 0.24, *p* < 0.001; *φ_Binding_* = 0.08, *p* < 0.01). The communality of the complexity requirements (i.e., binding) was incrementally predictive of WMC, over and above the communality of the basal speed requirement. Both standardized regression weights were significantly larger than zero (*p* < 0.001).

## 4. Discussion

The purpose of this study was to address the task complexity moderation of the relationship of mental speed with cognitive ability. To this end, we applied a theory-driven task complexity manipulation in line with the binding hypothesis of WMC on a variety of elementary cognitive tasks. We tested whether the proposed WMC requirements in terms of binding could be modeled above mental speed and whether these increased task requirements were incrementally predictive of WMC. In the next sections, we summarize and discuss our findings and address their implications.

### 4.1. Complexity Manipulations

For our study, we administered three different classes of choice–reaction-time tasks that are frequently used as elementary cognitive tasks measuring mental speed (Danthiir et al. 2012; Luck and Vogel 1997; Rouder et al. 2011). In order to pursue our research questions, we first manipulated task complexity, which can be understood in terms of WMC requirements (e.g., Larson et al. 1988). Although the administered task classes differ in their administration, their basic cognitive requirements are comparable. In addition, all tasks (and thus their trials) consist of a certain number of stimuli to be compared either with another set of stimuli (S–S bindings) or with an a priori defined response mapping (S–R bindings). Hence, successfully working on these tasks requires participants to build, update, and maintain temporary bindings of stimuli as specified in the binding account of WMC (Oberauer 2005a, 2019). We manipulated task complexity in line with the binding hypothesis by increasing the number of task-relevant S–S or S–R bindings that participants needed to maintain in an active state while performing the tasks. These complexity manipulations were achieved by increasing the set sizes of the tasks from 2 stimuli to 4 and 6 stimuli, respectively. As suggested by previous studies (Schmitz and Wilhelm 2016), we manipulated complexity within tasks in order to isolate the manipulated requirement. By applying the same theory-driven manipulations on all task classes, we ensured that the indicators of task performance could be interpreted meaningfully and independently of baseline differences between the tasks. Furthermore, employing different indicators and modeling heir communality by means of SEM helps reduce task impurity and specificity (Stahl et al. 2014).

We hypothesized that increased binding requirements offer a tractable and broadly applicable operationalization of task complexity. In line with predictions, this manipulation resulted in slower response times, lower accuracies, and lower diffusion model drift rates. Although the task classes might have inherently differed in their difficulty in the baseline conditions, we observed arguably more effortful information processing with increasing binding requirements across all tasks. In fact, the complexity manipulations were effective in all indicators of task performance. The present results are compatible with the binding hypothesis of working memory (Oberauer 2005a, 2019; Wilhelm et al. 2013) because the experimental manipulation increased the extent to which the tasks tapped WMC. This was especially apparent in the most complex task conditions of set size 6. Compared to the baseline conditions of the tasks with set size 2, the condition of set size 6 showed the most substantial effects.

### 4.2. Disentangling Tasks Requirements

We predicted that the applied complexity manipulations would allow for a targeted isolation of speed and WMC requirements. Of these, speed of processing (Barrouillet et al. 2011) and binding (Oberauer 2005a; 2019) were here investigated in depth. The basic idea was that the baseline condition of the ECTs would be a relatively pure speed measure, comparable to typical speed tasks (e.g., Danthiir et al. 2012; Schmitz and Wilhelm 2016). This baseline condition would be simple and exhibit relatively low error rates. In contrast, the tasks with increased binding requirements would be more complicated. This would be reflected in more effortful processing, i.e., reduced diffusion model drift rates, resulting in slower response times and somewhat reduced accuracies. Although the more complex tasks should have made it more challenging for participants to come to a correct solution quickly, these tasks should have still kept their basic characteristics as measures of mental speed.

The presented results confirm our expectations. Disentangling the task requirements of the simple and complex tasks by means of a confirmatory bi-factor analysis, we established two latent factors. The basal speed requirements of all tasks were captured with a general factor, and the experimentally manipulated binding requirements were captured with a nested factor. While the general speed factor reflected individual differences in speed, the binding factor reflected individual differences in binding capacity, above and beyond speed. By using 12 indicators, 6 simple tasks and 6 complex tasks, we ensured an appropriate breadth of the latent factors. In addition to that, substantial loadings across all indicators indicated sufficient variances in the indicators. This was also true for the identified factors, which displayed a reliable share of variance across specific tasks.

These results clearly favor the notion of a theory-driven complexity manipulation in ECTs. Furthermore, our modeling approach replicated previous research, where diffusion model drift rates were used as indicators for latent factors (e.g., Ratcliff et al. 2010, 2011; Schmiedek et al. 2007; Schmitz and Wilhelm 2016). We were also able to show that this parameter is suited for investigating individual differences in tasks with binary decision format.

The experimental procedure used in this study corresponds to the core notion of the recent process overlap theory (Kovacs and Conway 2016; 2019), namely that cognitive ability is constituted by a set of diverse executive processes (Oberauer 2009). In line with the literature (e.g., Kyllonen and Christal 1990) and the process overlap theory (Kovacs and Conway 2016, 2019), where complexity refers to the extent to which a test taps underlying executive processes, we claim that task complexity can be conceptualized in terms of the number and nature of supervisory or executive cognitive operations required to solve a task. Given our results, we argue that the experimental manipulation increased the extent to which the tasks tapped binding as an underlying executive process. This was particularly salient in the most complex task conditions of set size six. Compared to the baseline conditions of the tasks with set size 2, the condition of set size 6 showed the strongest effects for the complexity manipulations.

### 4.3. Relations with Cognitive Ability

As the binding requirements were manipulated in each ECT, this should have constituted an analogous increase in WMC requirements. In fact, this manipulation gave rise to a communality, which could be modeled as a nested specific factor across task classes. Lastly, we hypothesized that this WMC-related nested factor would be incrementally predictive of cognitive ability over and above the general speed factor. This would reflect expectations from the complexity hypothesis, which states that the correlation between the performance on ECTs and cognitive ability increases with task complexity (Sheppard and Vernon 2008; Vernon and Jensen 1984). In our case, the increased complexity of the administered tasks was modeled by a nested factor additionally to a basal speed factor.

We found that the nested factor, capturing the communality of the elevated binding requirements, was incrementally predictive of cognitive ability, over and above basal speed requirements of the tasks. Predictions from both factors were substantial, and both factors clearly explained distinct portions of variance in WMC. This can be interpreted as support of the complexity hypothesis. It is important to note that this does not only refer to empirical difficulty (as reflected in lower drift rates), but also to an increase in complexity in line with binding theory of WMC. Taken together, binding requirements in ECTs were shown to determine the relation with cognitive ability and, thereby, offer a more satisfactory account of task complexity.

Given that we separated two cognitive processes (i.e., basal speed and binding requirements), and both processes predicted unique shares of variance in WMC, one could argue that our results support the notion of process overlap theory. However, our results could also be interpreted as indicating that binding affordances are a key ingredient to WMC. Although we found complexity requirements to be more strongly related to WMC (which could be seen as a proxy for *g*), we also found that the basal speed requirements still explained almost as much unique variance in WMC as the incremental binding requirements. Thus, the general finding of the literature that more complex tasks exhibit higher loadings on *g* is certainly true, but this does not inevitably mean that binding is just one out of many executive processes jointly accounting for WMC. Therefore, we chose to be agnostic towards the idea of a reflective *g* factor.

### 4.4. Desiderata for Future Research

The sample of this study comprised young adults with an above-average level of education. Although we found substantial variances in and communalities across all indicators we cannot rule out range restriction, which would have attenuated the relations between the proposed latent factors on the predictor side and WMC on the criterion side. Future studies should replicate the theory-driven complexity manipulations in line with a WMC account with more heterogeneous samples. If range restriction adversely affects factor loadings and factor variances, the magnitude of the relations with the WMC factor might in fact increase.

In this study, we used WMC as a content-free proxy of cognitive ability. Although previous research has shown that WMC is fundamentally related to reasoning ability and fluid intelligence (e.g., Kane et al. 2005; Oberauer et al. 2005), future studies should complement the picture by using more and possibly other factors of cognitive ability.

Additionally, it should be noted that the binding hypothesis is not the only account suggested as an explanation of WMC. Other theory-driven accounts might also offer an interesting account for approaching task complexity and substituting it with a more meaningful notion. Other prominent accounts of WMC include, e.g., executive attention (e.g., Engle 2002; Miyake and Friedman 2012) and an interplay of primary and secondary memory (e.g., Unsworth and Engle 2006, 2007). Future research should, therefore, investigate the complexity hypothesis from the perspective of these accounts in order to further our understanding regarding task complexity in ECTs. However, these competing accounts of WMC might not transfer to manipulations of complexity as easily as the binding account.

## 5. Conclusions

This study contributes to the introduction of an empirically founded perspective of complexity in elementary cognitive tasks. We showed that complexity can be understood in terms of working memory capacity requirements. The binding hypothesis of working memory offered an especially practical account of approaching the notion of manipulating task complexity in a meaningful and theory-driven way. Our results support the literature in showing that the predictive validity of elementary cognitive tasks for cognitive ability indeed hinges on the complexity of these tasks. Furthermore, this study successfully combined an experimental manipulation of complexity in ECTs with bi-factor CFA modeling in order to separate relevant functions underlying cognitive ability. Taken together, binding costs account for a large amount of variance in cognitive ability over and above mental speed.

## Figures and Tables

**Table 1:** Descriptive statistics for speed tasks with mean response times (and standard deviations) in milliseconds, mean compound scores, diffusion model parameters, and effect sizes (Cohen’s *d*) across stimuli set sizes (2 vs. 4 vs. 6) within tasks.

Tasks		Set Size	*M*_RT_	*M*_err_	*a*	*v*	*Ter*	*d*_RT_ (95%-CI)	*d*_err_ (95%-CI)
***Change detection***	Color	2	665 (135)	0.08 (0.06)	0.13 (0.03)	0.21 (0.06)	0.38 (0.07)		
		4	843 (210)	0.15 (0.06)	0.13 (0.03)	0.15 (0.05)	0.49 (0.09)	1.00 [0.73; 1.28]	1.10 [0.82; 1.38]
		6	949 (253)	0.25 (0.07)	0.13 (0.03)	0.09 (0.04)	0.53 (0.12)	1.35 [1.06; 1.64]	2.60 [2.24; 2.95]
	Letter	2	563 (97)	0.04 (0.03)	0.13 (0.03)	0.27 (0.06)	0.32 (0.05)		
		4	729 (135)	0.09 (0.06)	0.14 (0.03)	0.19 (0.05)	0.40 (0.07)	1.42 [1.13; 1.71]	0.96 [0.68; 1.23]
		6	981 (224)	0.16 (0.08)	0.16 (0.03)	0.12 (0.03)	0.50 (0.10)	2.43 [2.09; 2.77]	1.88 [1.56; 2.19]
***Comparison***	Figure	2	954 (160)	0.05 (0.04)	0.15 (0.03)	0.22 (0.05)	0.64 (0.09)		
		4	1307 (248)	0.07 (0.06)	0.19 (0.04)	0.16 (0.04)	0.78 (0.13)	1.70 [1.40; 2.00]	0.31 [0.05; 0.57]
		6	1739 (402)	0.11 (0.08)	0.21 (0.05)	0.11 (0.03)	1 (0.20)	2.57 [2.22; 2.92]	0.88 [0.61; 1.16]
	Number	2	767 (101)	0.03 (0.03)	0.12 (0.02)	0.31 (0.06)	0.58 (0.06)		
		4	1072 (221)	0.07 (0.04)	0.15 (0.03)	0.19 (0.04)	0.70 (0.11)	1.78 [1.47; 2.10]	0.99 [0.72; 1.27]
		6	1563 (316)	0.06 (0.04)	0.21 (0.04)	0.14 (0.03)	0.91 (0.18)	3.40 [2.99; 3.80]	0.85 [0.57; 1.12]
	***M_compound_***								
***Substitution***	Figure	2	500 (89)	0.01 (0.01)	0 (0.59)	-	-		
		4	634 (87)	0.01 (0.02)	0 (0.60)	-	-	1.52 [1.22; 1.81]	0.24 [0; 0.50]
		6	748 (115)	0.02 (0.02)	0 (0.66)	-	-	2.40 [2.06; 2.74]	0.56 [0.30; 0.83]
	Letter	2	486 (90)	0.01 (0.02)	0 (0.67)	-	-		
		4	703 (116)	0.04 (0.03)	0 (0.67)	-	-	2.08 [1.76; 2.41]	0.99 [0.72; 1.27]
		6	854 (147)	0.03 (0.03)	0 (0.66)	-	-	3.01 [2.64; 3.40]	0.80 [0.53; 1.07]

*Note. M *= mean; *RT* = response time; *a* = response criterion of the diffusion model; *v* = drift rate of the diffusion model; *Ter* = non-decision time of the diffusion model; *d* = Cohen’s d (Cohen 1969). Standard deviations are given in parentheses.

**Table 2:** Standardized loadings of the models.

		Measurement Model (Figure 2)		Structural Model (Figure 3)		
		*λ* (*SE*)		*λ* (*SE*)		
Condition	Indicator	Speed	Binding	Speed	Binding	WMC
simple	CMP.fig	0.66 (0.06)	-	0.66 (0.06)	-	-
	CMP.num	0.63 (0.07)	-	0.63 (0.07)	-	-
	CDT.let	0.84 (0.04)	-	0.84 (0.04)	-	-
	CDT.col	0.73 (0.05)	-	0.74 (0.05)	-	-
	SUB.let	0.52 (0.08)	-	0.52 (0.08)	-	-
	SUB.fig	0.45 (0.08)	-	0.45 (0.08)	-	-
complex	CMP.fig	0.60 (0.07)	0.20 (0.10)	0.60 (0.07)	0.29 (0.09)	-
	CMP.num	0.52 (0.08)	0.15 (0.11)	0.52 (0.08)	0.15 (0.11)	-
	CDT.let	0.43 (0.09)	0.18 (0.12)	0.40 (0.09)	0.36 (0.11)	-
	CDT.col	0.37 (0.09)	0.35 (0.12)	0.35 (0.09)	0.37 (0.11)	-
	SUB.let	0.47 (0.08)	0.44 (0.12)	0.47 (0.08)	0.38 (0.10)	-
	SUB.fig	0.35 (0.09)	0.53 (0.13)	0.35 (0.09)	0.36 (0.11)	-
	R1B.let	-	-	-	-	0.65 (0.09)
	R1B.num	-	-	-	-	0.50 (0.10)
	R1B.fig	-	-	-	-	0.64 (0.09)

*Note*. CMP.fig = comparison figural, CMP.num = comparison numerical, CDT.let = change detection letter, CDT.col = change detection color, SUB.let = substitution letter, SUB.fig = substitution figure, R1B.let = Recall−1-Back letter, R1B.num = Recall−1-Back number, R1B.fig = Recall−1-Back figure.

## Data Availability

Data and analyses scripts are provided in an online repository (OSF): https://osf.io/em3sg/, accessed on 16 March 2021.
